# Design, synthesis and biological evaluation of 1-Aryl-5-(4-arylpiperazine-1-carbonyl)-1*H*-tetrazols as novel microtubule destabilizers

**DOI:** 10.1080/14756366.2020.1759582

**Published:** 2021-01-31

**Authors:** Chao Wang, Yuelin Li, Zi Liu, Zeyu Wang, Zihan Liu, Shuai Man, Yujing Zhang, Kai Bao, Yingliang Wu, Qi Guan, Daiying Zuo, Weige Zhang

**Affiliations:** a Key Laboratory of Structure-Based Drug Design and Discovery, Ministry of Education, Shenyang Pharmaceutical University, Shenyang, China; b Department of Pharmacology, Shenyang Pharmaceutical University, Shenyang, China; c Wuya College of Innovation, Shenyang Pharmaceutical University, Shenyang, China

**Keywords:** Tetrazole, microwave, antiproliferative activity, microtubule destabilizer, molecular docking

## Abstract

A series of 1-aryl-5-(4-arylpiperazine-1-carbonyl)-1*H*-tetrazols as microtubule destabilizers were designed, synthesised and evaluated for anticancer activity. Based on bioisosterism, we introduced the tetrazole moiety containing the hydrogen-bond acceptors as B-ring of XRP44X analogues. The key intermediates ethyl 1-aryl-1*H*-tetrazole-5-carboxylates **10** can be simply and efficiently prepared *via* a microwave-assisted continuous operation process. Among the compounds synthesised, compound **6–31** showed noteworthy potency against SGC-7901, A549 and HeLa cell lines. In mechanism studies, compound **6–31** inhibited tubulin polymerisation and disorganised microtubule in SGC-7901 cells by binding to tubulin. Moreover, compound **6–31** arrested SGC-7901cells in G2/M phase. This study provided a new perspective for development of antitumor agents that target tubulin.

## 1. Introduction

**Figure 1.**
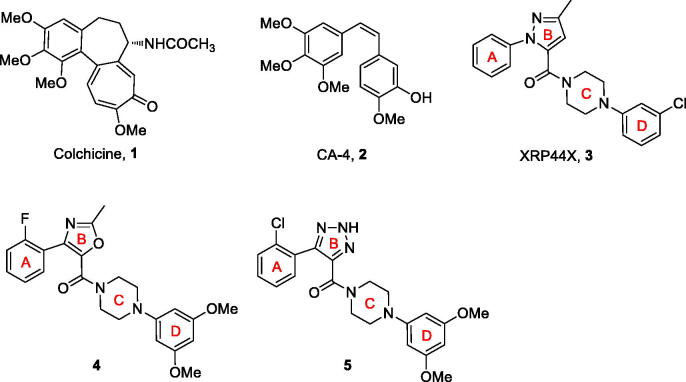
Chemical structures of some microtubule destabilizers.

Microtubule, is considered an important target for anticancer drug discovery, playing a crucial role in a wide range of fundamental cell functions including the shape maintenance, intracellular transport and cell division^1^. Microtubule-targeted agents, according to the mechanism of interfering with microtubule dynamics, have been classified into microtubule stabilisers (taxanes and epothilones) and microtubule destabilizers (alkaloids and colchicine (**1**))^2^. Given the extensively successful clinical use of vinca alkaloids, the microtubule destabilizers have aroused great interest among medicinal chemists^3^. Over the past decades, a great many outstanding microtubule destabilizers have been reported, such as combretastatin A-4 (CA-4, **2**)^4^ and XRP44X (**3**)^5^ (Figure 1).

XRP44X, an arylpyrazole derivative, was developed by Wasylyk et al. as a novel microtubule destabilizer, which prominently inhibited the polymerisation process of tubulin into microtubules by interacting with the colchicine-binding site of tubulin and displayed potent cytotoxic activities against a wide variety of human cancer cell lines at low nanomolar concentrations. A series of aryloxazole derivatives have been synthesised and were found to be potent inhibitors of tubulin polymerisation by Pae and co-workers. Among them, compound **4** showed excellent antiproliferative *in vitro* and effectively reduced tumour growth *in vivo* using a tumour xenograft model^6^. Our groups discovered a series of aryltriazole derivatives as a result of structural modifications of the lead compound XRP44X^7^. The most potent compound 5 exhibited excellent antiproliferative activity *via* disrupting cytoskeleton.

As shown in Figure 1, XRP44X and its analogues molecules can be divided into three major structural elements i.e., A-ring (substituted phenyl), B-ring (five-membered heterocycle, such as pyrazole, oxazole and 1,2,3-triazole), C-ring (piperazine), D-ring (substituted phenyl), and a carbonyl linkage between B-ring and C-ring. Bioisosterism plays a major role in the search for analogues of an active drug molecule. Application of bioisosterism instead of the B-ring on the structure of XRP44X is one of the strategies often used in design of the XRP44X analogues^6^. The modification of B-ring has led to varied cytotoxic activity.

In the last decade, there has been great interest in compounds containing the 1*H*-tetrazol scaffold because of its unique chemical structure and broad spectrum of biological properties including anticancer activity. For example, the 1-(3,4,5-Trimethoxyphenyl)-5-(4-ethoxyphenyl)-1*H*-tetrazole was designed as tubulin inhibitor, which showed low IC_50_ values at nanomolar level^8^.

**Figure 2.**
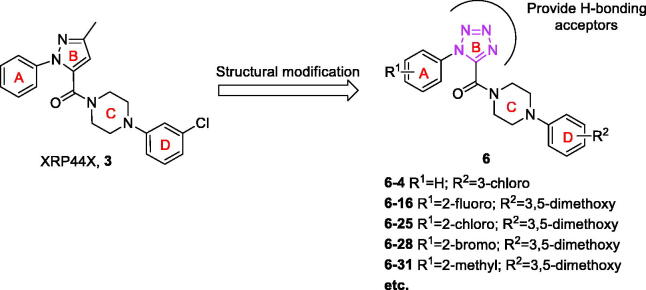
Design of the target compounds.

As a part of our continuing effort on the development of novel antitumor agents, we designed a series of novel XRP44X analogues by introducing an 1*H*-tetrazol, hydrogen-bonding acceptors, as B-ring of XRP44X (Figure 2). Herein, we described the detailed synthetic routes, antiproliferative, tubulin polymerisation, analysis of immunofluorescence staining and cell cycle analysis of these compounds.

## 2. Result and discussion

### 2.1. Chemistry

**Scheme 1.**
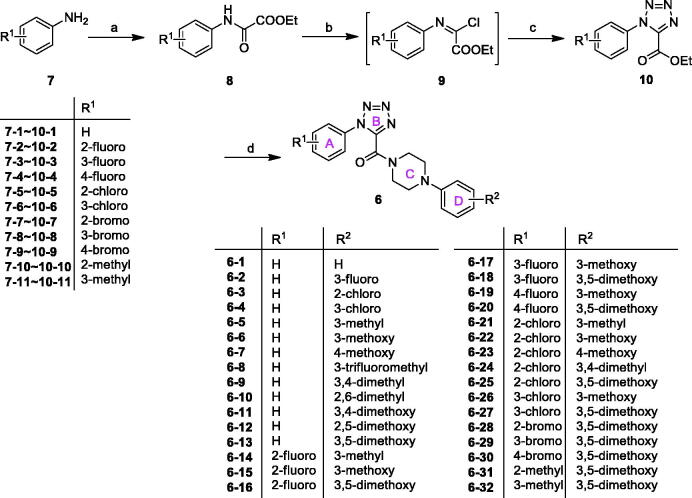
Reagents and conditions: (a) EtO_2_CCOCl, Et_3_N, DCM, rt., 1 h; (b) Ph_3_P, CCl_4_, 130 °C, MW, 20 min; (c) NaN_3_, MeCN, rt., overnight; (d) arylpiperazines, AlMe_3_ (1.0 M solution in heptane), DCM, rt., overnight.

The general synthetic approach for the preparation of 1-aryl-5-(4-arylpiperazine-1-carbonyl)-1*H*-tetrazol (**6**) was illustrated in Scheme 1. First of all, the substituted aromatic amines (**7**) were reacted with ethyl oxalate in dichloromethane to afford corresponding intermediates (**8**) at room temperature^9^^,^^10^. Subsequently, **8** were reacted with triphenylphosphine and carbon tetrachloride to give corresponding (*E*)-ethyl 2-chloro-2-(arylimino)acetates (**9**) *via* Appel reaction under microwave irradiation. Without further purification, compounds **9** reacted with sodium azide in acetonitrile to get the key intermediates ethyl 1-aryl-1*H*-tetrazole-5-carboxylates (**10**) in a continuous operation process^11–13^. Finally, the key intermediates **10** reacted with corresponding arylpiperazines to afford the target compounds (**6**) in the presence of trimethylaluminium^14^.

Under conventional heating conduction, the reaction of **8** with PPh_3_ in CCl_4_ to generate (*E*)-ethyl 2-chloro-2-(arylimino)acetates (**9**) suffered from long reaction time and low yields (Table 1, entries 1–5). Microwave irradiation offered many advantages, such as rate enhancements and higher yields, over conventional heating and had become a popular technique that was widely used in organic synthesis today^15–18^. When microwave irradiation was incorporated into synthesis of **9**–**8**, we found that the reaction rate was greatly improved. We systematically screened the influence of reaction temperature and time on the yield of **9**–**8** under microwave conditions. As shown in Table 1, the optimised condition was confirmed to be microwave irradiation at 130 °C for 20 min (entry 8). Accordingly, all intermediates **9** were obtained smoothly.

**Table 1.** The optimisation of conditions for the preparation of compound **9–8**.

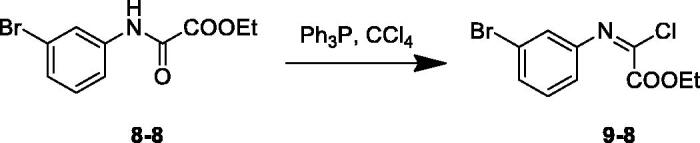				
Entry	Heating	Temp (∘C)	Time (min)	Yield (%)
1^a^	Oil bath	Reflux	60	Trace
2^a^	Oil bath	Reflux	360	10^c^
3^a^	Oil bath	Reflux	600	30^c^
4^a^	Oil bath	Reflux	720	41^c^
5^a^	Oil bath	Reflux	960	52^d^
6^b^	MW	120	20	45^c^
7^b^	MW	130	10	50^d^
**8** ^b^	**MW**	**130**	**20**	**67** ^d^
9^b^	MW	130	30	62^d^
10^b^	MW	140	20	60^d^

^a^ Reaction conditions: ethyl 2-((3-bromophenyl)amino)-2-oxoacetate (1 mmol), Ph3P (1.5 mmol), CCl4 (5 ml).

^b^ Reaction conditions: ethyl 2-((3-bromophenyl)amino)-2-oxoacetate (1 mmol), Ph3P (1.5 mmol), CCl4 (5 ml), MW.

^c^ The yields were determined by HPLC.

^d^ Isolated yields.

### 2.2. Biological evaluation

#### 2.2.1. *In vitro* antiproliferative activity

All the target compounds have been evaluated for *in vitro* the antiproliferative activities against different three human cancer cell lines (gastric adenocarcinoma SGC-7901 cells, lung adenocarcinoma A549 cells and cervical carcinoma HeLa cells) using MTT assay with colchicine and CA-4 as references. To examine the more detailed structure-activity relationships, modifications towards A-ring and D-ring were performed.

Careful observation of Table 2 revealed that introduction of substituent into the ortho-position of A-ring could remarkably enhance the antiproliferative effect, such as **6**–**28** ∼ **6**–**30**. The overall preference order of substituent at the ortho-position of A-ring is as follows: 2-methyl > 2-fluoro > 2-chloro > H. Furthermore, in the case of D-ring 3,5-dimethoxyphenyl fused compounds showed significant anticancer activities. Compound **6–31** was found to be the most potent compound among all the target compounds with IC50 value of 0.090–0.650 μM against the three cancer cell lines.

**Table 2.** The antiproliferative activities of all target compounds

Compound	*R* ^1^	*R* ^2^	(IC_50_ ± SD, μM)^a^		
			SGC-7901	A549	HeLa
**6–1**	H	H	>30	9.92 **±** 0.46	>30
**6–2**	H	3-fluoro	28.3 **±** 2.1	>30	>30
**6–3**	H	2-chloro	>30	>30	>30
**6–4**	H	3-chloro	13.8 **±** 0.8	8.22 **±** 0.23	18.2 **±** 1.8
**6–5**	H	3-methyl	27.8 **±** 2.0	25.3 **±** 1.9	22.1 **±** 1.6
**6–6**	H	3-methoxy	15.5 **±** 1.2	13.2 **±** 1.0	19.3 **±** 1.7
**6–7**	H	4-methoxy	>30	>30	>30
**6–8**	H	3-trifluoromethyl	16.1 **±** 1.4	15.0 **±** 1.2	20.6 **±** 1.8
**6–9**	H	3,4-dimethyl	>30	>30	>30
**6–10**	H	2,6-dimethyl	>30	>30	>30
**6–11**	H	3,4-dimethoxy	>30	27.3 **±** 1.9	>30
**6–12**	H	2,5-dimethoxy	2.47 **±** 0.21	3.86 **±** 0.27	3.34 **±** 0.23
**6–13**	H	3,5-dimethoxy	1.68 **±** 0.12	3.10 **±** 0.25	3.11 **±** 0.19
**6–14**	2-fluoro	3-methyl	17.2 **±** 1.4	5.80 **±** 0.30	>30
**6–15**	2-fluoro	3-methoxy	11.3 **±** 0.9	10.0 **±** 1.1	26.1 **±** 2.3
**6–16**	2-fluoro	3,5-dimethoxy	0.504 **±** 0.021	2.17 **±** 0.20	0.360 **±** 0.017
**6–17**	3-fluoro	3-methoxy	29.8 **±** 3.0	20.9 **±** 1.5	28.7 **±** 3.2
**6–18**	3-fluoro	3,5-dimethoxy	4.2 **±** 0.24	6.71 **±** 0.19	1.01 **±** 0.05
**6–19**	4-fluoro	3-methoxy	>30	>30	27.6 **±** 2.7
**6–20**	4-fluoro	3,5-dimethoxy	12.2 **±** 1.6	>30	10.5 **±** 0.9
**6–21**	2-chloro	3-methyl	4.59 **±** 0.18	10.5 **±** 0.41	10.6 **±** 0.47
**6–22**	2-chloro	3-methoxy	11.8 **±** 0.50	11.7 **±** 0.49	>30
**6–23**	2-chloro	4-methoxy	>30	>30	>30
**6–24**	2-chloro	3,4-dimethyl	24.3 **±** 1.6	>30	22.0 **±** 1.5
**6–25**	2-chloro	3,5-dimethoxy	1.08 **±** 0.13	3.12 **±** 0.15	0.580 **±** 0.023
**6–26**	3-chloro	3-methoxy	>30	>30	29.4 **±** 0.6
**6–27**	3-chloro	3,5-dimethoxy	1.51 **±** 0.09	7.77 **±** 0.17	1.85 **±** 0.13
**6–28**	2-bromo	3,5-dimethoxy	2.53 **±** 0.11	>30	5.67 **±** 0.19
**6–29**	3-bromo	3,5-dimethoxy	4.73 **±** 0.14	>30	7.48 **±** 0.18
**6–30**	4-bromo	3,5-dimethoxy	>30	29.4 **±** 3.1	>30
**6–31**	2-methyl	3,5-dimethoxy	**0.090 ± 0.008**	**0.650 ± 0.017**	**0.268 ± 0.012**
**6–32**	3-methyl	3,5-dimethoxy	1.44 **±** 0.09	2.34 **±** 0.16	0.812 **±** 0.034
**CA–4** ^b^			0.036 **±** 0.002	0.070 **±** 0.007	0.034 **±** 0.004
**Colchicine** ^b^			0.096 ± 0.009	0.075 ± 0.011	0.066 ± 0.005

^a^ IC_50_: the half maximal inhibitory concentration.

^b^ Used as positive controls.

#### 2.2.2. Effect on tubulin polymerisation

**Figure 3.**
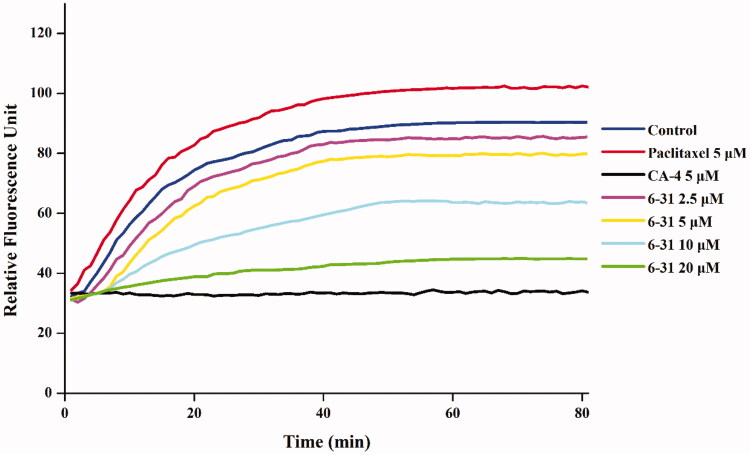
The effect of compound **6**–**31** on tubulin polymerisation. The tubulin had been pre-incubated for 1 min with **6**–**31** at 2.5 µM, 5 µM, 10 µM and 20 µM, CA-4 at 5 µM, Paclitaxel at 5 µM or vehicle DMSO at room temperature before GTP was added to start the tubulin polymerisation reactions. The reaction was monitored at 37 °C.

In order to examine whether the compounds interact with tubulin, we chose the most active compound **6**–**31** to evaluate for its inhibition of tubulin polymerisation *in vitro*. Microtubule polymerisation inhibitor (CA-4) and microtubule stabilising agent (Paclitaxel) were used as the positive and negative controls, respectively. As shown in Figure 3, compound **6**–**31** inhibited tubulin assembly in a concentration-dependent manner. In contrast, paclitaxel could raise the proportion of tubulin polymerisation in comparison with the untreated cells. The results suggested that compound **6**–**31** interferes with the microtubule polymerisation in a similar way as CA-4.

#### 2.2.3. Analysis of immunofluorescence staining

**Figure 4.**
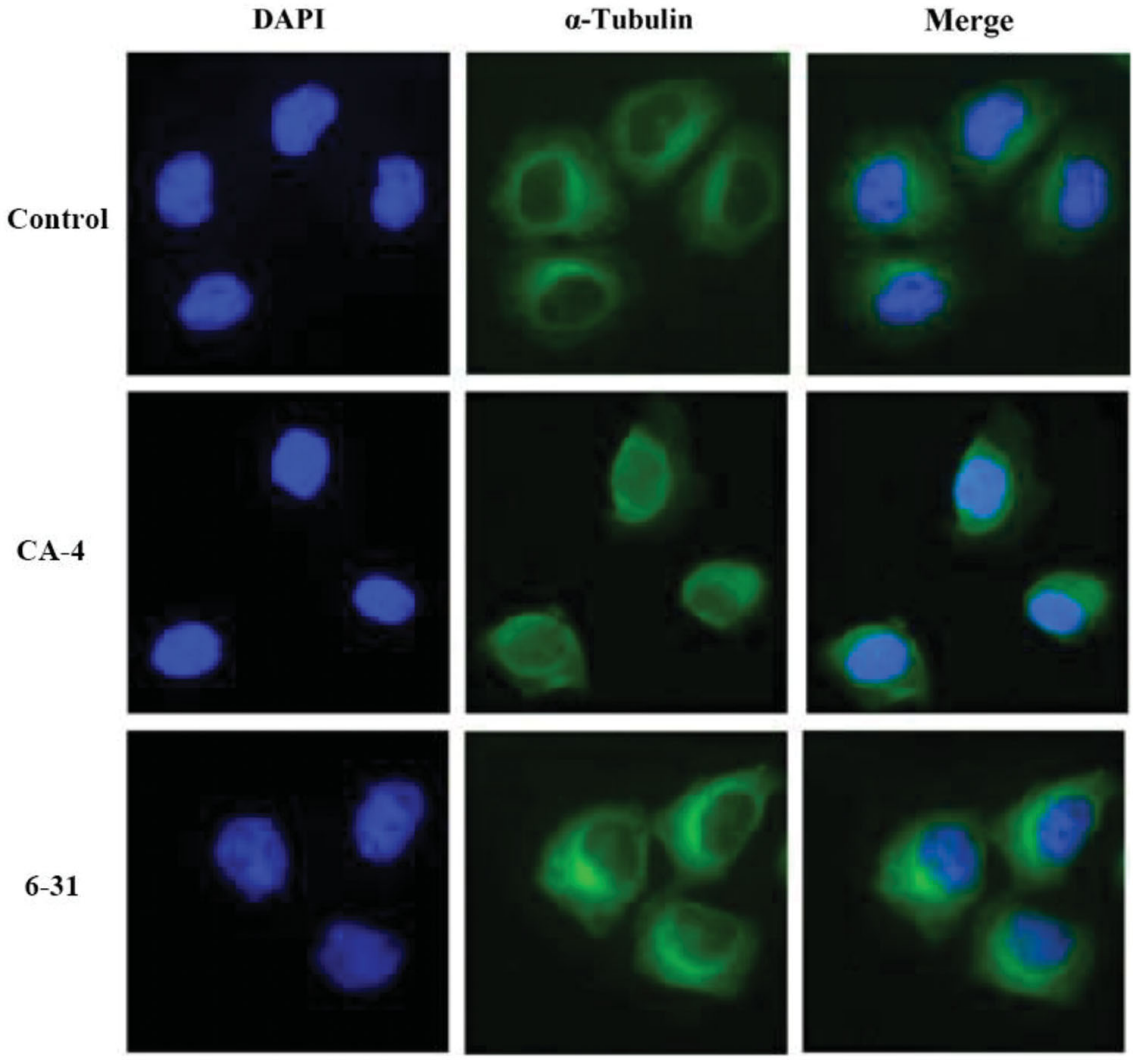
Effects of **6**–**31** and CA-4, at their respective two-fold IC_50_ concentrations, on the cellular microtubule network and microtubule reassemble by immunofluorescence. SGC-7901 cells were treated with **6**–**31** or CA-4 for 24 h, and then direct microscopy detection of the fixed and stained cell was performed. The cellular microtubules were stained with anti-a-tubulin-FITC specific antibodies (green). DNA was stained by 4,6-diamidino-2-phenylindole (DAPI, blue). (For interpretation of the references to colour in this figure legend, the reader is referred to the Web version of this article).

To further confirm the influence of inhibition of tubulin polymerisation in cells, immunofluorescence staining was carried out. SGC-7901 cell lines were treated for 24 h with CA-4 and compound **6**–**31**, at their respective 2-fold IC_50_ concentrations. As given in Figure 4, the microtubule network without drug treatment displays normal arrangement and organisation in control cells. Whereas SGC-7901 cells were treated with CA-4 and compound **6**–**31** and the results demonstrated that microtubules were destroyed and wrapped around the nucleus in comparison with the control. These results suggest that compound **6**–**31** inhibits microtubule assembly and disrupts cytoskeleton similarly to CA-4.

#### 2.2.4. Cell cycle analysis

It is well known that most tubulin inhibitors induce cell cycle arrest in the G2/M phase^7^. Thus, the effect of compound **6–31**on the cell cycle of SGC-7901 cells was analysed by flow cytometry. SGC-7901 cells were incubated with CA-4 or compound **6–31**, at their respective two-fold IC_50_ concentrations, and the proportion of tested cells at different cell cycle phases was analysed by flow cytometry after 0, 12, 24, 36, 48, and 72 h of treatment, respectively.

**Figure 5.**
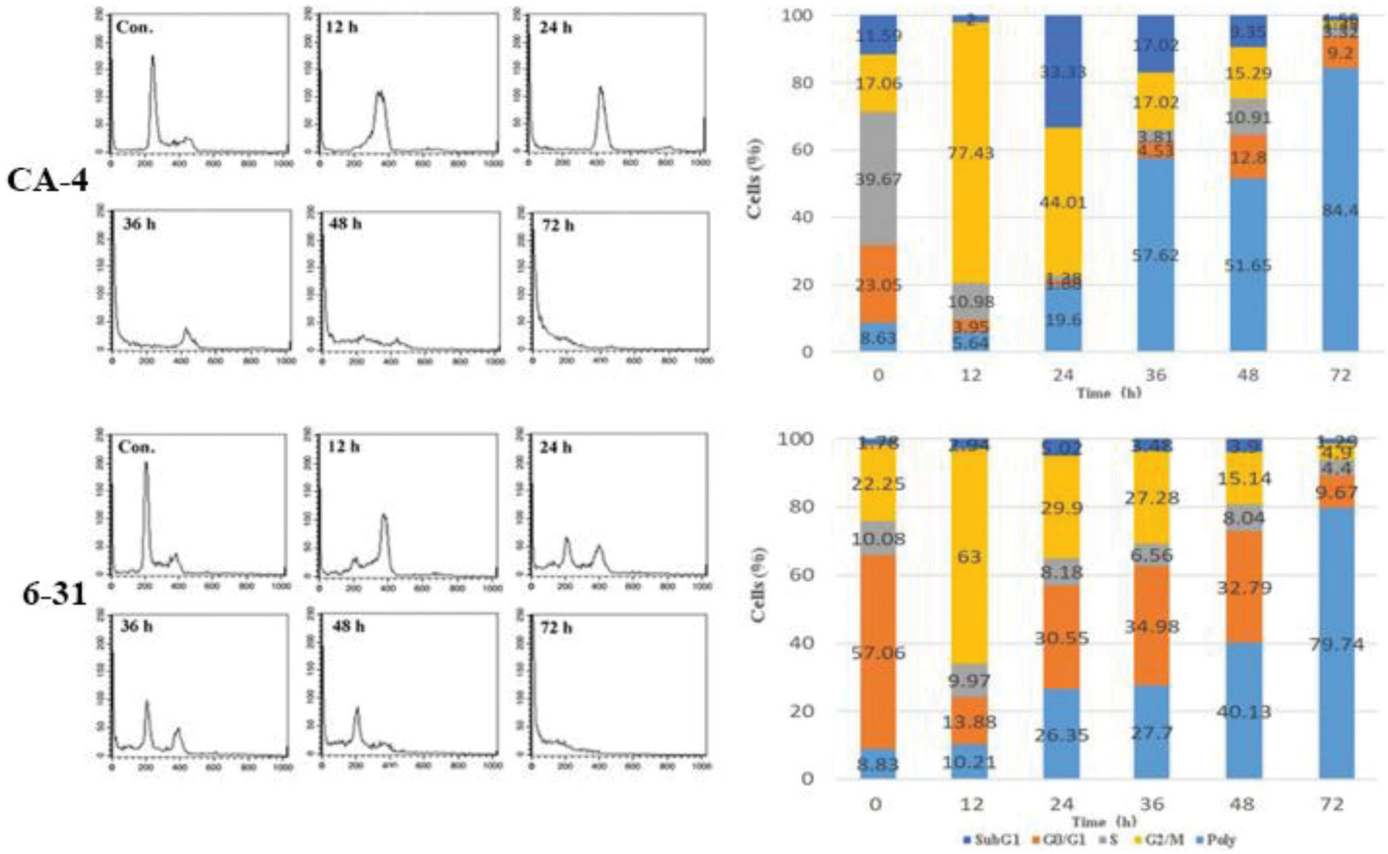
The effect of CA-4 and compound **6**–**31** on cell cycle. SGC-7901 cells lines treated with CA-4 and compound **6**–**31**, at their respective two-fold IC_50_ concentrations, for 0, 12, 24, 36, 48 and 72 h.

As shown in Figure 5, cells treated with either CA-4 or compound **6–31** were arrested at the G2/M phase. In addition, whereas CA-4 or **6–31** induced a sharply decrease of G1 cell population, and an increase of G2/M cell population in time-dependent manner. After treatment with compound **6–31** for 12 h, the percentage of cells in the G2/M phase increased to 63.0% from 22.2%. After 72 h, there were significantly increased numbers of cells in sub-G1 after exposure to CA-4 and compound **6–31**, respectively, indicating the induction of apoptosis. The cell cycle analysis suggests that compound **6–31** arrests SGC-7901 cell in G2/M phase followed by cellular apoptosis.

### 2.3. Molecular modelling

**Figure 6.**
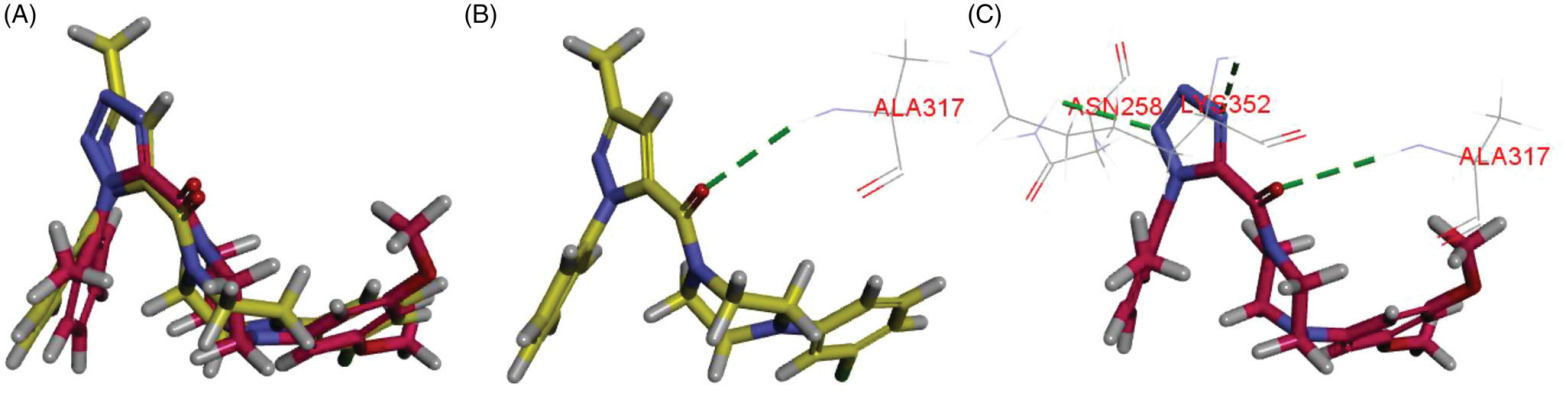
(A) Superimposition of XRP44X (yellow) and **6**–**31** (red) in colchicine-binding site; (B) interaction of XRP44X (yellow) with tubulin (green dotted lines represent H-bond); (C) Interactions of **6**–**31** (red) with tubulin (green dotted lines represent H-bond).

To further understand the binding interactions, molecular docking of the most active compound **6–31** was carried out with tubulin crystal structure (PDB code: 3HKC) using the CDOCKER programme of Discovery Studio 3.0 software. As shown in Figure 6, XRP44X and compound **6–31** are located at the same position with a similar conformation in the binding pocket. Meanwhile, the hydrogen bond exists between the carbonyl group of XRP44X or compound **6–31** with amino acid residue Alaβ317. Moreover, it is worth noting that the residue of Asnβ258 forms a hydrogen bond with the 2 *N* of 1*H*-tetrazole of compound **6–31** and the residue of Lysβ352 forms a hydrogen bond with the 4 *N* of 1*H*-tetrazole of compound **6–31**. It suggests that the 1*H*-tetrazole derivatives can not only maintain the right conformation, but also nicely nestle in the active site. The 1*H*-tetrazole moiety and unique active site interactions set the stage for structure-based design of more potent derivatives.

### 2.4. Prediction of drug-like properties

Furthermore, to explore the drug-like properties of the target compounds, some physicochemical properties of XRP44X and compound **6–31** were predicted using free online website (http://www.swissadme.ch/index.php) for their adaptability with Lipinski’s rule of five^19^. As shown in Table 3, XRP44X and compound **6–31** conform well to the Lipinski’s rule of five. Compared with XRP44X, compound **6–31** has a lower value of lipid-water partition coefficient. This data indicates that compound **6–31** may have better water solubility than XRP44X. In addition, compound **6–31** exists six hydrogen bond receptors, much more than XRP44X, which helps to reduce the binding energy between the compound and the action site.

**Table 3.** Prediction of physicochemical properties^a^ of **3** and **6–31.**

Compound	miLogP	TPSA	natoms	MW	nON	nOHNH	nrotb
Standard	<5	<140		<500	<10	<5	≤10
**3**	3.48	41.37	48	380.87	2	0	4
**6–31**	2.29	85.61	54	408.45	6	0	6

^a^ miLogP: molinspiration predicted Log P; TPSA: topological polar surface area; natoms: No. of atoms; MW: molecular weight; nON: No. of hydrogen bond acceptors; nOHNH: No. of hydrogen bond donors; nrotb: No. of rotatable bonds.

## 3. Conclusion

In summary, we had designed and synthesised a series of XRP44X derivatives having a 1*H*-tetrazole B-ring as the hydrogen-bonding acceptors and found that these compounds showed good growth inhibition activities against a range of human cancer cells. Among them, compound **6–31**, represented the most active compound with IC_50_ values of 0.090**–**0.650 μM against three cancer cell lines. Moreover, compound **6–31** could disrupt microtubule network in living cancer cells, arrest cell cycle at G2/M phase and induce apoptosis in a dose- and time-dependent manner. Docking studies suggest that compound **6–31** may be a potential tubulin inhibitor. A hydrogen bond was present between Alaβ317 with the carbonyl group of compound **6–31**. Another two hydrogen bonds were also observed between Asnβ258 and Lysβ352 with the 1*H*-tetrazole (B-ring). In addition, the prediction of drug-like properties studies shows that compound **6–31** has better pharmacokinetic properties than XRP44X. All of these results indicate compound **6–31** as a promising microtubule destabilizer for further investigation in anticancer drug development.

## 4. Experimental

### 4.1. Chemistry

#### 4.1.1. Materials and methods

All of reagents and solvents were purchased from chemical company. ^1^H NMR and ^13 ^C NMR spectra were tested in CDCl_3_ with TMS as the internal reference on a Bruker AVANCE 400 or 600 (^1^H at 400 or 600 MHz, ^13 ^C at 150 MHz). Mass spectra (MS) were measured on an Agilent 1100-sl mass spectrometer with an electrospray ionisation source from Agilent Co. Ltd. High resolution accurate mass determinations (HRMS) for all of the final target compounds were obtained on a Bruker Micromass Time of Flight mass spectrometer equipped with electrospray ionisation (ESI). TLC analysis was used for determining the extent of reactions under UV light (wavelength: 365 nm and 254 nm). Melting point was measured (uncorrected) on hot-stage microscope (Beijing Taike, X-4). The microwave reactions were carried out in a single mode cavity microwave synthesiser (CEM Corporation, NC, USA).

#### 4.1.2. General synthetic procedures for arylpiperazines

A solution of arylamine (1 mmol), bis(2- chloroethyl)amine hydrochloride (1.1 mmol) and K_2_CO_3_ (3 mmol) in *n*-BuOH were stirred at irradiated in a microwave reactor for 30 min at 150 °C. The reaction mixture was cooled to room temperature and dissolved in methanol (4 ml), followed by the addition of diethyl ether (150 ml). The precipitate formed was recovered by filtration and washed with diethyl ether to obtain arylpiperazine as HCl salt. The HCl salt was used for the next reaction without further purification^20^^,^^21^.

#### 4.1.3. General synthetic procedures for ethyl 2-oxo-2-(arylamino)acetates (8)

To a solution of substituted aniline (10 mmol) and triethylamine (1 ml, 10 mmol) in DCM was added ethyl chlorooxoacetate (10 mmol) in DCM. The reaction mixture was stirred for 1 h at room temperature. The reaction mixture was poured into water and extracted with DCM (50 ml × 3). The combined organic layer was washed with brine, dried over anhydrous Na_2_SO_4_, filtered and concentrated to yield the crude product. The crude product was purified by column chromatography (*n*-hexane/EtOAc = 1:2) on silica gel to afford pure products. For example:

##### 4.1.3.1. Ethyl 2-oxo-2-(phenylamino)acetate (8–1)

White Solid; yield: 91%; MS (ESI) *m/z* 194.1 [M + H]^+^.

##### 4.1.3.2. Ethyl 2-((3-bromophenyl)amino)-2-oxoacetate (8–8)

White Solid; yield: 92%; MS (ESI) *m/z* 272.0 [M + H]^+^.

#### 4.1.4. General synthetic procedures for (E)-ethyl 2-chloro-2-(arylimino)acetates (9)

A solution of triphenylphosphine (1.5 mmol) and ethyl 2-oxo-2-(arylamino)acetate (1 mmol) in CCl_4_ (5 ml) were stirred at irradiated in a microwave reactor for 20 min at 130 °C. The reaction mixtures were cooled to room temperature and the precipitate was filtered off. The filtrate was concentrated to yield the crude product. The crude product was purified by column chromatography (*n*-hexane/EtOAc = 4:1) on silica gel to afford pure products. For example:

##### 4.1.4.1. (E)-Ethyl 2-((3-bromophenyl)imino)-2-chloroacetate (9–8)

Light yellow oil; yield: 67%; ^1^H NMR (600 MHz, CDCl_3_): *δ =*  7.65 (1H, s), 7.63 (1H, d, *J* = 1.4 Hz), 7.22 (1H, t, *J* = 2.9 Hz), 7.16 (1H, t, *J* = 2.0 Hz), 4.40 (2H, q, *J* = 7.2 Hz), 1.38 (3H, t, *J* = 7.1 Hz). ESI-MS: *m/z* = 290.5 [M + H]^+^.

#### 4.1.5. General synthetic procedures for ethyl 1-aryl-1H-tetrazole-5-carboxylates (10)

Because of the instability of intermediates **9**, we described a microwave-assisted continuous operation process method rather than stepwise operation process for the conversion of **8**–**10**. A solution of triphenylphosphine (1.5 mmol) and ethyl 2-oxo-2-(arylamino)acetate **8** (1 mmol) in CCl_4_ (5 ml) were stirred at irradiated in a microwave reactor for 20 min at 130 °C. The reaction mixtures were cooled to room temperature and the precipitate was filtered off. The filtrate was evaporated and dissolved in CH_3_CN. Sodium azide (1.2 mmol) was added at room temperature under N_2_ for 16 h. The solvent was then removed under reduced pressure and extracted with ethyl acetate (50 ml × 3). The combined organic layer was washed with water and brine and then dried over Na_2_SO_4_, filtered and concentrated to yield the crude product. The crude product was purified by column chromatography (*n*-hexane/EtOAc = 3:1) on silica gel to afford pure products. For example:

##### 4.1.5.1. Ethyl 1-phenyl-1H-tetrazole-5-carboxylate (10–1)

Light yellow Solid; yield: 76%; ^1^H NMR (600 MHz, CDCl_3_): *δ* = 7.70 (3H, m), 7.60 (2H, m), 4.55 (2H, q), 1.47 (3H, t, *J* = 6.8 Hz). ESI-MS: *m/z* = 219.1 [M + H]^+^.

##### 4.1.5.2. Ethyl 1-(2-fluorophenyl)-1H-tetrazole-5-carboxylate (10–2)

Yellow Solid; yield: 66%; ^1^H NMR (600 MHz, CDCl_3_): *δ* = 7.63 (1H, m), 7.57 (1H, m), 7.40 (1H, t, *J* = 7.2 Hz), 7.34 (1H, m), 4.46 (2H, q), 1.37 (3H, t, *J* = 6.8 Hz). ESI-MS: *m/z* = 237.1 [M + H]^+^.

##### 4.1.5.3. Ethyl 1–(3-fluorophenyl)-1H-tetrazole-5-carboxylate (10–3)

Light yellow Solid; yield: 71%; ^1^H NMR (600 MHz, CDCl_3_): *δ* = 7.57 (1H, m), 7.34 (2H, m), 7.29 (1H, m), 4.47 (2H, q), 1.40 (3H, t, *J* = 8.1 Hz). ESI-MS: *m/z* = 237.0 [M + H]^+^.

##### 4.1.5.4. Ethyl 1-(4-fluorophenyl)-1H-tetrazole-5-carboxylate (10–4)

Light yellow Solid; yield: 77%; ^1^H NMR (600 MHz, CDCl_3_): *δ* = 7.51 (2H, m), 7.27 (2H, m), 4.56 (2H, q), 1.40 (3H, t, *J* = 7.1 Hz). ESI-MS: *m/z* = 237.1 [M + H]^+^.

##### 4.1.5.5. Ethyl 1-(2-chlorophenyl)-1H-tetrazole-5-carboxylate (10–5)

Yellow Solid; yield: 70%; ^1^H NMR (600 MHz, CDCl_3_): *δ* = 7.61 (2H, m), 7.51 (2H, m), 4.42 (2H, q), 1.34 (3H, t, *J* = 7.1 Hz). ESI-MS: *m/z* = 253.0 [M + H]^+^.

##### 4.1.5.6. Ethyl 1-(3-chlorophenyl)-1H-tetrazole-5-carboxylate (10–6)

Yellow Solid; yield: 73%; ^1^H NMR (600 MHz, CDCl_3_): *δ* = 7.60 (1H, m), 7.54 (2H, m), 7.42 (1H, m), 4.47 (2H, q), 1.40 (3H, t, *J* = 7.5 Hz). ESI-MS: *m/z* = 253.0 [M + H]^+^.

##### 4.1.5.7. Ethyl 1-(2-bromophenyl)-1H-tetrazole-5-carboxylate (10–7)

Yellow Solid; yield: 76%; ^1^H NMR (600 MHz, CDCl_3_): *δ* = 7.64 (1H, d, *J* = 7.8 Hz), 7.35 (1H, t, *J* = 7.4 Hz), 7.11 (1H, d, *J* = 8.4 Hz), 6.92 (1H, d, *J* = 7.8 Hz), 4.48 (2H, q), 1.45 (3H, t, *J* = 7.4 Hz). ESI-MS: *m/z* = 297.0 [M + H]^+^.

##### 4.1.5.8. Ethyl 1-(3-bromophenyl)-1H-tetrazole-5-carboxylate (10–8)

Light yellow Solid; yield: 62%; ^1^H NMR (600 MHz, CDCl_3_): *δ* = 7.76 (1H, m), 7.70 (1H, m), 7.46 (2H, m), 4.47 (2H, q), 1.40 (3H, t, *J* = 7.4 Hz). ESI-MS: *m/z* = 297.0 [M + H]^+^.

##### 4.1.5.9. Ethyl 1-(4-bromophenyl)-1H-tetrazole-5-carboxylate (10–9)

Light yellow Solid; yield: 69%; ^1^H NMR (600 MHz, CDCl_3_): *δ* = 7.72 (2H, d, *J* = 8.5 Hz), 7.40 (2H, d, *J* = 8.5 Hz), 4.46 (2H, q), 1.40 (3H, t, *J* = 7.81 Hz). ESI-MS: *m/z* = 297.0 [M + H]^+^.

##### 4.1.5.10. Ethyl 1-(o-tolyl)-1H-tetrazole-5-carboxylate (10–10)

Yellow Solid; yield: 67%; ^1^H NMR (600 MHz, CDCl_3_): *δ* = 7.52 (1H, m), 7.42 (1H, d, *J* = 7.4 Hz), 7.39 (1H, t, *J* = 7.7 Hz), 7.25 (1H, dd, *J* = 1.2 Hz, *J* = 7.7 Hz), 4.40 (2H, q), 2.05 (1H, s), 1.32 (3H, t, *J* = 6.8 Hz). ESI-MS: *m/z* = 233.1 [M + H]^+^.

##### 4.1.5.11. Ethyl 1-(m-tolyl)-1H-tetrazole-5-carboxylate (10–11)

Yellow Solid; yield: 75%; ^1^H NMR (600 MHz, CDCl_3_): *δ* = 7.45 (1H, t, *J* = 8.4 Hz), 7.41 (1H, d, *J* = 8.1 Hz), 7.28 (2H, t, *J* = 11.8 Hz), 4.45 (2H, q), 2.46 (3H, s), 1.37 (3H, t, *J* = 7.3 Hz). ESI-MS: *m/z* = 233.1 [M + H]^+^.

#### 4.1.6. General synthetic procedures for 1-aryl-5–(4-arylpiperazine-1-carbonyl)-1H-tetrazols (6)

To a solution of arylpiperazine (0.1 mmol) in anhydrous DCM was added trimethylaluminum (0.5 ml, 1 M in heptane). The reaction was stirred at room temperature under N_2_ for 15 min. A solution of an appropriate ethyl 1-aryl-1*H*-tetrazole-5-carboxylate (0.1 mmol) in anhydrous DCM was added and the reaction was stirred at room temperature under N_2_ for 16 h. The reaction was quenched with 5 ml of 1 M HCl and diluted with DCM. The combined organic layer was washed with water and brine and then dried over Na_2_SO_4_, filtered and concentrated to yield the crude product. The crude product was purified by column chromatography (*n*-hexane/EtOAc = 1:1) on silica gel to afford pure products.

##### 4.1.6.1. 1-Phenyl-5–(4-phenylpiperazine-1-carbonyl)-1H-tetrazol (6–1)

White Solid; yield: 45%; Mp: 140.0–141.8 °C; ^1^H NMR (600 MHz, CDCl_3_): *δ* = 7.50 (5H, d, *J* = 10.8 Hz), 7.22 (2H, t, *J* = 7.6 Hz), 6.85 (3H, m), 3.86 (2H, s), 3.63 (2H, s), 3.17 (2H, s), 3.07 (2H, s). ^13 ^C NMR (150 MHz, CDCl_3_): *δ* = 154.6, 149.4, 146.6, 132.7, 129.6, 128.8 (2 C), 128.3 (2 C), 122.9 (2 C), 120.1, 115.9 (2 C), 48.9, 48.4, 45.9, 41.5; HRMS calcd for C_18_H_18_N_6_NaO [M + Na]^+^ 357.1440, found 357.1477.

##### 4.1.6.2. 1-Phenyl-5-(4-(3-fluorophenyl)piperazine-1-carbonyl)-1H-tetrazol (6–2)

Light yellow Solid; yield: 49%; Mp: 135.9–137.8 °C; ^1^H NMR (600 MHz, CDCl_3_): *δ* = 7.50 (5H, d), 7.14 (1H, q, *J* = 7.8 Hz), 6.58 (1H, q, *J* = 8.5 Hz), 6.51 (2H, m,), 3.84 (2H, t, *J* = 4.7 Hz), 3.64 (2H, t, *J* = 4.4 Hz), 3.17 (2H, t, *J* = 5.0 Hz), 3.08 (2H, t, *J* = 4.7 Hz). ^13 ^C NMR (150 MHz, CDCl_3_): *δ* = 162.7 (d, *J* = 237.7 Hz), 154.6, 151.0 (d, *J* = 9.2 Hz), 146.6, 132.7, 129.7, 129.4 (d, *J* = 10.2 Hz), 128.8 (2 C), 123.0 (2 C), 110.9 (d, *J* = 2.5 Hz), 106.2 (d, *J* = 20.4 Hz), 102.6 (d, *J* = 24.0 Hz), 48.3, 47.8, 45.7, 41.3; HRMS calcd for C_18_H_17_FN_6_NaO [M + Na]^+^ 375.1346, found 375.1378.

##### 4.1.6.3. 1-Phenyl-5-(4-(2-chlorophenyl)piperazine-1-carbonyl)-1H-tetrazol (6–3)

Light yellow Solid; yield: 60%; Mp: 76.7–78.4 °C; ^1^H NMR (600 MHz, CDCl_3_): *δ* = 7.59 (5H, m), 7.01 (4H, m), 3.96 (2H, t, *J* = 4.2 Hz), 3.68 (2H, t, *J* = 4.7 Hz), 3.10 (2H, t, *J* = 5.0 Hz), 3.02 (2H, t, *J* = 4.9 Hz). ^13 ^C NMR (150 MHz, CDCl_3_): *δ* = 154.8, 147.0, 146.7, 129.7, 129.7, 128.8 (2 C), 128.6, 126.7, 123.7, 122.87 (2 C), 119.6, 114.3, 50.3, 49.8, 46.4, 41.9; HRMS calcd for C_18_H_17_ClN_6_NaO [M + Na]^+^ 391.1050, found 391.1064.

##### 4.1.6.4. 1-Phenyl-5-(4-(3-chlorophenyl)piperazine-1-carbonyl)-1H-tetrazol (6–4)

Light yellow Solid; yield: 58%; Mp: 102.5–104.0 °C; ^1^H NMR (600 MHz, CDCl_3_): *δ* = 7.58 (5H, m), 7.19 (1H, t, *J* = 8.1 Hz), 6.87 (2H, t, *J* = 8.5 Hz), 6.77 (1H, dd, *J* = 2.0 Hz, *J* = 8.1 Hz), 3.91 (2H, t, *J* = 5.0 Hz), 3.71 (2H, t, *J* = 5.0 Hz), 3.25 (2H, t, *J* = 4.8 Hz), 3.16 (2H, t, *J* = 5.4 Hz). ^13 ^C NMR (150 MHz, CDCl_3_): *δ* = 154.6, 150.5, 146.6, 134.1, 132.6, 129.7, 129.3, 128.8 (2 C), 123.0 (2 C), 119.7, 115.7, 113.7, 48.4, 47.8, 45.7, 41.3; HRMS calcd for C_18_H_17_ClN_6_NaO [M + Na]^+^ 391.1050, found 391.1073.

##### 4.1.6.5. 1-Phenyl-5-(4-(3-methylphenyl)piperazine-1-carbonyl)-1H-tetrazol (6–5)

White Solid; yield: 61%; Mp: 135.2–137.1 °C; ^1^H NMR (600 MHz, CDCl_3_): *δ* = 7.49 (5H, s), 7.10 (1H, s), 6.66 (3H, s), 3.85 (2H, s), 3.61 (2H, s), 3.15 (2H, s), 3.05 (2H, s), 2.25 (3H, s). ^13 ^C NMR (150 MHz, CDCl_3_): *δ* = 154.6, 149.5, 146.7, 138.1, 132.7, 129.7, 128.8 (2 C), 128.1, 122.9 (2 C), 120.9, 116.8, 113.0, 48.9, 48.5, 46.0, 41.5, 20.7; HRMS calcd for C_19_H_20_N_6_NaO [M + Na]^+^ 371.1596, found 371.1633.

##### 4.1.6.6. 1-Phenyl-5-(4-(3-methoxyphenyl)piperazine-1-carbonyl)-1H-tetrazol (6–6)

White Solid; yield: 67%; Mp: 144.3–146.2 °C; ^1^H NMR (600 MHz, CDCl_3_): *δ* = 7.57 (5H, d, *J* = 7.5 Hz), 7.20 (1H, t, *J* = 8.4 Hz), 6.50 (2H, m), 6.44 (1H, s), 3.92 (2H, t, *J* = 4.8 Hz), 3.7 (3H, s), 3.69 (2H, t, *J* = 5.1 Hz), 3.24 (2H, t, *J* = 4.9 Hz), 3.14 (2H, t, *J* = 4.8 Hz). ^13 ^C NMR (150 MHz, CDCl_3_): *δ* = 159.6, 154.6, 150.8, 146.6, 132.7, 129.7, 129.0, 128.8 (2 C), 123.0 (2 C), 108.5, 104.6, 102.4, 54.2, 48.8, 48.3, 45.9, 41.5; HRMS calcd for C_19_H_20_N_6_NaO_2_ [M + Na]^+^ 387.1545, found 387.1578.

##### 4.1.6.7. 1-Phenyl-5-(4-(4-methoxyphenyl)piperazine-1-carbonyl)-1H-tetrazol (6–7)

White Solid; yield: 71%; Mp: 152.7–154.2 °C; ^1^H NMR (600 MHz, CDCl_3_): *δ* = 7.51 (5H, m), 6.79 (4H, t, *J* = 13.5 Hz) , 3.86 (2H,s), 3.71 (3H, s), 3.62 (2H,s), 3.05 (2H, s), 2.95 (2H,s). ^13 ^C NMR (150 MHz, CDCl_3_): *δ* = 154.6, 153.7, 146.7, 143.7, 132.7, 129.6, 128.8 (2 C), 122.9 (2 C), 118.2 (2 C), 113.6 (2 C), 54.5, 50.3, 49.9, 46.1, 41.6; HRMS calcd for C_19_H_20_N_6_NaO_2_ [M + Na]^+^ 387.1545, found 387.1574.

##### 4.1.6.8. 1-Phenyl-5-(4-(3-trifluoromethyl)piperazine-1-carbonyl)-1H-tetrazol (6–8)

Light yellow Solid; yield: 60%; Mp: 77.7–79.2 °C; ^1^H NMR (600 MHz, CDCl_3_): *δ* = 7.59 (5H, m), 7.39 (1H, t, *J* = 7.8 Hz), 7.16 (1H, d, *J* = 7.8 Hz), 7.11 (1H, s), 7.06 (1H, d, *J* = 8.8 Hz), 3.95 (2H, t, *J* = 5.0 Hz), 3.76 (2H, t, *J* = 5.0 Hz), 3.30 (2H, t, *J* = 5.4 Hz), 3.22 (2H, t, *J* = 5.4 Hz). ^13 ^C NMR (150 MHz, CDCl_3_): *δ* = 154.6, 149.6, 146.5, 132.7, 129.7, 128.8, 128.8 (2 C), 128.7, 123.0 (2 C), 118.7, 116.3 (m), 114.2, 112.1 (m), 48.4, 47.9, 45.8, 41.4; HRMS calcd for C_19_H_17_F_3_N_6_NaO [M + Na]^+^ 425.1314, found 425.1311.

##### 4.1.6.9. 1-Phenyl-5-(4-(3,4-dimethylphenyl)piperazine-1-carbonyl)-1H-tetrazol (6–9)

Pink Solid; yield: 57%; Mp: 138.6–140.1 °C; ^1^H NMR (600 MHz, CDCl_3_): *δ* = 7.57 (5H, m), 7.04 (1H, d, *J* = 8.1 Hz) , 6.73 (1H, s), 6.66 (1H, d, *J* = 8.4 Hz) , 3.29 (2H, t, *J* = 4.7 Hz), 3.68 (2H, s), 3.18 (2H, t, *J* = 5.4 Hz), 3.07 (2H, t, *J* = 4.7 Hz), 2.23 (3H, s), 2.19 (2H, s). ^13 ^C NMR (150 MHz, CDCl_3_): *δ* = 154.6, 147.6, 146.7, 136.5, 129.7, 129.3, 128.8 (2 C), 128.7, 122.9 (2 C), 117.9, 114.3, 113.6, 49.3, 49.0, 46.1, 41.6, 19.1, 17.8; HRMS calcd for C_20_H_22_N_6_NaO [M + Na]^+^ 385.1753, found 385.1788.

##### 4.1.6.10. 1-Phenyl-5-(4-(2,6-dimethylphenyl)piperazine-1-carbonyl)-1H-tetrazol (6–10)

Light yellow Solid; yield: 49%; Mp: 71.7–73.2 °C; ^1^H NMR (600 MHz, CDCl_3_): *δ* = 7.55 (5H, m), 7.08 (2H, d, *J* = 8.1 Hz), 6.83 (1H, t, *J* = 8.1 Hz), 3.74 (2H, s), 3.60 (2H, t, *J* = 4.0 Hz), 2.90 (2H, t, *J* = 5.4 Hz), 2.85 (2H, t, *J* = 4.7 Hz), 1.65 (6H, s). ^13 ^C NMR (150 MHz, CDCl_3_): *δ* = 169.2, 159.7, 150.7, 131.0, 130.7, 129.3 (2 C), 126.9 (2 C), 124.4 (2 C), 119.9 (2 C), 115.3, 52.0, 51.6, 46.9, 42.0, 17.4 (2 C); HRMS calcd for C_20_H_22_N_6_NaO [M + Na]^+^ 385.1753, found 385.1788.

##### 4.1.6.11. 1-Phenyl-5-(4-(3,4-dimethoxyphenyl)piperazine-1-carbonyl)-1H-tetrazol (6–11)

Light yellow Solid; yield: 53%; Mp: 101.6–102.6 °C; ^1^H NMR (600 MHz, CDCl_3_): *δ* = 7.59 (5H, d, *J* = 10.8 Hz), 7.80 (1H, d, *J* = 9.5 Hz), 6.55 (1H, s), 6.44 (1H, d, *J* = 8.1 Hz), 3.94 (2H, t, *J* = 6.1 Hz), 3.87 (3H, s), 3.84 (3H, s), 3.70 (2H, t, *J* = 5.4 Hz), 3.14 (2H, t, *J* = 5.1 Hz), 3.05 (2H, t, *J* = 5.5 Hz). ^13 ^C NMR (150 MHz, CDCl_3_): *δ* = 154.6, 148.5, 146.6, 144.2, 143.5, 132.7, 129.7, 128.8 (2 C), 122.9 (2 C), 110.7, 107.9, 102.6, 55.2, 54.8, 50.4, 50.1, 46.2, 41.7; HRMS calcd for C_20_H_22_N_6_NaO_3_ [M + Na]^+^ 417.1651, found 417.1661.

##### 4.1.6.12. 1-Phenyl-5-(4-(2,5-dimethoxyphenyl)piperazine-1-carbonyl)-1H-tetrazol (6–12)

Light yellow Solid; yield: 51%; Mp: 78.9–80.8 °C; ^1^H NMR (600 MHz, CDCl_3_): *δ* = 7.58 (5H, m), 6.79 (1H, d, *J* = 9.1 Hz), 6.53 (1H, dd, *J* = 2.5 Hz, *J* = 9.4 Hz), 6.45 (1H, d, *J* = 3.2 Hz), 3.94 (2H, t, *J* = 5.0 Hz), 3.82 (3H, s), 3.76 (3H, s), 3.66 (2H, t, *J* = 4.7 Hz), 3.10 (2H, t, *J* = 5.0 Hz), 2.99 (2H, t, *J* = 5.0 Hz). ^13 ^C NMR (150 MHz, CDCl_3_): *δ* = 154.7, 153.0, 146.8, 145.4, 139.9, 132.7, 129.6, 128.8 (2 C), 122.8 (2 C), 110.9, 105.6, 105.2, 54.8, 54.6, 49.7, 49.1, 46.3, 41.7; HRMS calcd for C_20_H_22_N_6_NaO_3_ [M + Na]^+^ 417.1651, found 417.1693.

##### 4.1.6.13. 1-Phenyl-5-(4-(3,5-dimethoxyphenyl)piperazine-1-carbonyl)-1H-tetrazol (6–13)

Light yellow Solid; yield: 61%; Mp: 121.3–123.2 °C; ^1^H NMR (600 MHz, CDCl_3_): *δ* = 7.57 (5H, m), 6.07 (3H, m), 3.91 (2H, t, *J* = 4.9 Hz), 3.77 (6H, s), 3.69 (2H, t, *J* = 4.7 Hz), 3.23 (2H, t, *J* = 4.9 Hz), 3.13 (2H, t, *J* = 5.0 Hz). ^13 ^C NMR (150 MHz, CDCl_3_): *δ* = 160.5 (2 C), 154.6, 151.3, 146.6, 132.6, 129.7, 128.8 (2 C), 122.9 (2 C), 94.8 (2 C), 91.6, 54.3 (2 C), 48.8, 48.3, 45.8, 41.4; HRMS calcd for C_20_H_23_N_6_O_3_ [M + H]^+^ 395.1832, found 395.1859.

##### 4.1.6.14. 1-(2-Fluorophenyl)-5-(4-(3-methylphenyl)piperazine-1-carbonyl)-1H-tetrazol (6–14)

Light yellow Solid; yield: 69%; Mp: 102.5–104.0 °C; ^1^H NMR (600 MHz, CDCl_3_): *δ* = 7.70 (1H, m), 7.56 (1H, m), 7.39 (1H, t, *J* = 8.1 Hz), 7.28 (1H, t, *J* = 9.5 Hz), 7.19 (1H, t, *J* = 8.1 Hz), 6.77 (3H, t, *J* = 6.7 Hz), 4.03 (2H, t, *J* = 4.7 Hz), 3.92 (2H, t, *J* = 4.7 Hz), 3.27 (4H, t, *J* = 5.1 Hz), 2.34 (3H, s). ^13 ^C NMR (150 MHz, CDCl_3_): *δ* = 153.9, 153.9 (d, *J* = 245.7 Hz), 149.6, 147.6, 131.3 (d, *J* = 8.1 Hz), 128.1, 125.9, 124.4 (d, *J* = 3.5 Hz), 123.1 (d, *J* = 7.1 Hz) 120.8, 116.7, 115.7 (d, *J* = 19.4 Hz), 114.7 (d, *J* = 18.4 Hz), 112.9, 48.9, 48.5, 46.2, 41.5, 20.7; HRMS calcd for C_19_H_19_FN_6_NaO [M + Na]^+^ 389.1502, found 389.1531.

##### 4.1.6.15. 1-(2-Fluorophenyl)-5-(4-(3-methoxyphenyl)piperazine-1-carbonyl)-1H-tetrazol (6–15)

Light yellow Solid; yield: 52%; Mp: 105.9–107.8 °C; ^1^H NMR (600 MHz, CDCl_3_): *δ* = 7.70 (1H, m), 7.56 (1H, m), 7.38 (1H, t, *J* = 7.6 Hz), 7.28 (1H, t, *J* = 9.8 Hz), 7.21 (1H, t, *J* = 8.7 Hz), 6.56 (1H, m), 6.49 (2H, d, *J* = 6.9 Hz), 4.02 (2H, t, *J* = 5.0 Hz), 3.91 (2H, t, *J* = 4.7 Hz), 3.80 (3H, s), 3.28 (4H, t, *J* = 5.0 Hz). ^13 ^C NMR (150 MHz, CDCl_3_): *δ* = 159.6, 153.9, 153.9 (d, *J* = 251.7 Hz), 150.9, 147.6, 131.4 (d, *J* = 8.6 Hz), 129.0, 125.9, 124.4 (d, *J* = 3.8 Hz), 121.3 (d, *J* = 12.5 Hz), 115.7 (d, *J* = 18.1 Hz), 108.5, 104.5, 102.3, 54.2, 48.7, 48.2, 46.1, 41.4; HRMS calcd for C_19_H_19_FN_6_NaO_2_ [M + Na]^+^ 405.1451, found 405.1493.

##### 4.1.6.16. 1-(2-Fluorophenyl)-5-(4-(3,5-dimethoxyphenyl)piperazine-1-carbonyl)-1H-tetrazol (6–16)

Yellow Solid; yield: 69%; Mp: 52.1–53.2 °C; ^1^H NMR (600 MHz, CDCl_3_): *δ* = 7.70 (1H, m), 7.56 (1H, m), 7.39 (1H, t, *J* = 7.8 Hz), 7.29 (1H, m), 6.11 (2H, d, *J* = 2.0 Hz), 6.08 (1H, t, *J* = 2.0 Hz), 4.02 (2H, t, *J* = 5.2 Hz), 3.90 (2H, t, *J* = 4.6 Hz), 3.79 (6H, s), 3.27 (4H, t, *J* = 5.2 Hz). ^13 ^C NMR (150 MHz, CDCl_3_): *δ* = 160.6 (2 C), 153.9, 153.9 (d, *J* = 253.8 Hz), 151.5, 147.6, 131.4 (d, *J* = 7.6 Hz), 126.0, 124.4 (d, *J* = 3.5 Hz), 121.3 (d, *J* = 12.2 Hz), 115.6 (d, *J* = 19.3 Hz), 94.8 (2 C), 91.5, 54.3 (2 C), 48.7, 48.2, 42.0, 41.4; HRMS calcd for C_20_H_22_FN_6_O_3_ [M + H]^+^ 413.1737, found 413.1768.

##### 4.1.6.17. 1-(3-Fluorophenyl)-5-(4-(3-methoxyphenyl)piperazine-1-carbonyl)-1H-tetrazol (6–17)

Light yellow Solid; yield: 64%; Mp: 124.3–126.0 °C; ^1^H NMR (600 MHz, CDCl_3_): *δ* = 7.54 (1H, m), 7.40 (2H, t, *J* = 8.1 Hz), 7.28 (1H, t, *J* = 8.8 Hz), 7.20 (1H, t, *J* = 9.4 Hz), 6.53 (1H, d, *J* = 8.3 Hz), 6.49 (1H, d, *J* = 7.6 Hz), 6.45 (1H, s), 3.94 (2H, t, *J* = 4.3 Hz), 3.79 (3H, s), 3.76 (2H, t, *J* = 5.0 Hz), 3.27 (2H, t, *J* = 5.8 Hz), 3.20 (2H, t, *J* = 4.7 Hz). ^13 ^C NMR (150 MHz, CDCl_3_): *δ* = 161.7 (d, *J* = 245.2 Hz), 159.6, 154.2, 150.7, 146.5, 133.7 (d, *J* = 9.2 Hz), 130.2 (d, *J* = 8.6 Hz), 129.1, 118.7 (d, *J* = 3.0 Hz), 116.8 (d, *J* = 20.9 Hz), 111.0 (d, *J* = 27.5 Hz),108.5, 104.7, 102.4, 54.2, 48.8, 48.3, 46.0, 41.6; HRMS calcd for C_19_H_19_FN_6_NaO_2_ [M + Na]^+^ 405.1451, found 405.1500.

##### 4.1.6.18. 1-(3-Fluorophenyl)-5-(4-(3,5-dimethoxyphenyl)piperazine-1-carbonyl)-1H-tetrazol (6–18)

Light yellow Solid; yield: 52%; Mp: 48.7–50.1 °C; ^1^H NMR (600 MHz, CDCl_3_): *δ* = 7.54 (1H, m), 7.40 (2H, m), 7.28 (1H, m), 6.08 (3H, s), 3.94 (2H, t, *J* = 5.3 Hz), 3.78 (8H, s), 3.26 (2H, t, *J* = 5.3 Hz), 3.20 (2H, t, *J* = 5.3 Hz). ^13 ^C NMR (150 MHz, CDCl_3_): *δ* = 162.6 (d, *J* = 255.9 Hz), 161.6 (2 C), 155.3, 152.3, 147.6, 134.8 (d, *J* = 10.2 Hz), 131.2 (d, *J* = 9.6 Hz), 119.8 (d, *J* = 3.0 Hz), 117.9 (d, *J* = 20.8 Hz), 112.1 (d, *J* = 26.5 Hz), 95.9 (2 C), 92.8, 55.3 (2 C), 49.9, 49.4, 46.9, 42.5; HRMS calcd for C_20_H_22_FN_6_O_3_ [M + H]^+^ 413.1737, found 413.1759.

##### 4.1.6.19. 1-(4-Fluorophenyl)-5-(4-(3-methoxyphenyl)piperazine-1-carbonyl)-1H-tetrazol (6–19)

White Solid; yield: 81%; Mp: 117.4–118.7 °C; ^1^H NMR (600 MHz, CDCl_3_): *δ* = 7.59 (2H, m), 7.25 (2H, m), 7.19 (1H, t, *J* = 8.4 Hz), 6.52 (1H, dd, *J* = 2.3 Hz, *J* = 8.4 Hz), 6.48 (1H, dd, *J* = 2.0 Hz, *J* = 8.4 Hz), 6.45 (1H, s), 3.91 (2H, t, *J* = 5.4 Hz), 3.78 (5H, m), 3.25 (2H, t, *J* = 5.4 Hz), 3.20 (3H, t, *J* = 5.0 Hz). ^13 ^C NMR (150 MHz, CDCl_3_): *δ* = 163.5 (d, *J* = 245.7 Hz), 160.7, 155.3, 151.8, 147.7, 130.1, 129.8 (d, *J* = 2.5 Hz), 126.5 (d, *J* = 8.1 Hz) (2 C), 116.9 (d, *J* = 23.5 Hz) (2 C), 109.5, 105.7, 103.4, 55.3, 49.9, 49.3, 47.1, 42.6; HRMS calcd for C_19_H_19_FN_6_NaO_2_ [M + Na]^+^ 405.1451, found 405.1482.

##### 4.1.6.20. 1-(4-Fluorophenyl)-5-(4-(3,5-dimethoxyphenyl)piperazine-1-carbonyl)-1H-tetrazol (6–20)

Brown Solid; yield: 45%; Mp: 48.1–49.4 °C; ^1^H NMR (600 MHz, CDCl_3_): *δ* = 7.59 (2H, m), 7.25 (2H, m), 6.08 (3H, s), 3.91 (2H, t, *J* = 4.7 Hz), 3.78 (8H, s), 3.25 (2H, t, *J* = 5.0 Hz), 3.20 (2H, t, *J* = 4.4 Hz). ^13 ^C NMR (150 MHz, CDCl_3_): *δ* = 162.5 (d, *J* = 247.7 Hz), 160.6 (2 C), 154.3, 151.3, 146.7, 128.8 (d, *J* = 4.6 Hz), 125.4 (d, *J* = 9.2 Hz) (2 C), 115.5 (d, *J* = 23.5 Hz) (2 C), 94.8 (2 C), 91.7, 54.3 (2 C), 48.9, 48.4, 45.9, 41.5; HRMS calcd for C_20_H_22_FN_6_O_3_ [M + H]^+^ 413.1737, found 413.1774.

##### 4.1.6.21. 1-(2-Chlorophenyl)-5-(4-(3-methylphenyl)piperazine-1-carbonyl)-1H-tetrazol (6–21)

Light yellow Solid; yield: 72%; Mp: 107.0–109.3 °C; ^1^H NMR (600 MHz, CDCl_3_): *δ* = 7.56 (4H, m), 7.19 (1H, t, *J* = 7.4 Hz), 6.76 (3H, m), 4.09 (2H, t, *J* = 4.75 Hz), 3.87 (2H, t, *J* = 5.4 Hz), 3.27 (2H, t, *J* = 5.4 Hz), 3.24 (2H, t, *J* = 5.4 Hz), 2.33 (3H, s). ^13 ^C NMR (150 MHz, CDCl_3_): *δ* = 153.6, 149.6, 148.0, 138.1, 131.1, 129.4, 129.4, 128.8, 128.1, 127.5, 127.1, 120.8, 116.7, 112.9, 48.9, 48.4, 46.2, 41.5, 20.7; HRMS calcd for C_19_H_19_ClN_6_NaO [M + Na]^+^ 405.1207, found 405.1246.

##### 4.1.6.22. 1-(2-Chlorophenyl)-5-(4-(3-methoxyphenyl)piperazine-1-carbonyl)-1H-tetrazol (6–22)

Light yellow Solid; yield: 74%; Mp: 99.8–101.5 °C; ^1^H NMR (600 MHz, CDCl_3_): *δ* = 7.56 (4H, m), 7.21 (1H, t, *J* = 9.1 Hz), 6.55 (1H, dd, *J* = 2.1 Hz, *J* = 8.4 Hz), 6.48 (2H, m), 4.09 (2H, t, *J* = 5.6 Hz), 3.87 (2H, t, *J* = 4.7 Hz), 3.80 (3H, s), 3.29 (2H, t, *J* = 5.2 Hz), 3.25 (2H, t, *J* = 4.7 Hz). ^13 ^C NMR (150 MHz, CDCl_3_): *δ* = 159.6, 153.6, 150.9, 148.0, 131.1, 131.0, 129.4, 129.0, 128.8, 127.5, 127.1, 108.4, 104.5, 102.3, 54.2, 48.8, 48.2, 46.1, 41.4; HRMS calcd for C_19_H_19_ClN_6_NaO_2_ [M + Na]^+^ 421.1156, found 421.1199.

##### 4.1.6.23. 1-(2-Chlorophenyl)-5-(4-(4-methoxyphenyl)piperazine-1-carbonyl)-1H-tetrazol (6–23)

Yellow Solid; yield: 68%; Mp: 119.9–121.2 °C; ^1^H NMR (600 MHz, CDCl_3_): *δ* = 7.56 (4H, m), 6.92 (2H, d, *J* = 8.8 Hz), 6.86 (2H, d, *J* = 9.5 Hz), 4.08 (2H, t, *J* = 4.7 Hz), 3.87 (2H, t, *J* = 4.7 Hz), 3.78 (3H, s), 3.16 (2H, t, *J* = 5.09 Hz), 3.12 (2H, t, *J* = 5.0 Hz). ^13 ^C NMR (150 MHz, CDCl_3_): *δ* = 153.6, 148.0 (2 C), 143.8, 131.1, 131.0, 129.4, 128.8, 127.5, 127.1, 118.1 (2 C), 113.5 (2 C), 54.5, 50.3, 49.7, 46.3, 41.6; HRMS calcd for C_19_H_19_ClN_6_NaO_2_ [M + Na]^+^ 421.1156, found 421.1197.

##### 4.1.6.24. 1-(2-Chlorophenyl)-5-(4-(3,4-dimethylphenyl)piperazine-1-carbonyl)-1H-tetrazol (6–24)

Light yellow Solid; yield: 73%; Mp: 110.9–112.0 °C; ^1^H NMR (600 MHz, CDCl_3_): *δ* = 7.55 (4H, m), 7.05 (1H, d, *J* = 8.8 Hz), 6.76 (1H, d, *J* = 2.7 Hz), 6.70 (1H, dd, *J* = 2.7 Hz, *J* = 8.4 Hz), 4.07 (2H, t, *J* = 5.0 Hz), 3.87 (2H, t, *J* = 4.7 Hz), 3.22 (2H, t, *J* = 5.4 Hz), 3.19 (2H, t, *J* = 5.0 Hz), 2.24 (3H, s), 2.19 (3H, s). ^13 ^C NMR (150 MHz, CDCl_3_): *δ* = 153.6, 148.0, 147.8, 136.4, 131.1, 131.0, 129.4, 129.3, 128.8, 128.7, 127.5, 127.1, 117.8, 113.5, 49.4, 48.8, 46.2, 41.6, 19.2, 17.8; HRMS calcd for C_20_H_21_ClN_6_NaO [M + Na]^+^ 419.1363, found 421.406.

##### 4.1.6.25. 1-(2-Chlorophenyl)-5-(4-(3,5-dimethoxyphenyl)piperazine-1-carbonyl)-1H-tetrazol (6–25)

Light yellow Solid; yield: 80%; Mp: 77.5–79.4 °C; ^1^H NMR (600 MHz, CDCl_3_): *δ* = 7.54 (4H, m), 6.09 (2H, s), 6.08 (1H, s), 4.08 (2H, t, *J* = 4.9 Hz), 3.86 (2H, t, *J* = 4.6 Hz), 3.78 (6H, s), 3.27 (2H, t, *J* = 4.6 Hz), 3.24 (2H, t, *J* = 5.5 Hz). ^13 ^C NMR (150 MHz, CDCl_3_): *δ* = 161.6 (2 C), 154.7, 152.5, 149.0, 132.2, 132.1, 130.4, 129.8, 128.5, 128.2, 95.8 (2 C), 92.6, 55.3 (2 C), 49.8, 49.2, 47.1, 42.5; HRMS calcd for C_20_H_21_ClN_6_NaO_3_ [M + Na]^+^ 451.1261, found 451.1268.

##### 4.1.6.26. 1-(3-Chlorophenyl)-5-(4-(3-methoxyphenyl)piperazine-1-carbonyl)-1H-tetrazol (6–26)

White Solid; yield: 81%; Mp: 150.8–152.4 °C; ^1^H NMR (600 MHz, CDCl_3_): *δ* = 7.63 (1H, s), 7.54 (1H, m), 7.50 (2H, d, *J* = 4.7 Hz), 7.20 (1H, t, *J* = 8.9 Hz), 6.53 (1H, d, *J* = 8.4 Hz), 6.49 (1H, d, *J* = 9.5 Hz), 6.45 (1H, s), 3.93 (2H, t, *J* = 5.4 Hz), 3.79 (5H, s), 3.27 (2H, t, *J* = 5.0 Hz), 3.21(2H, t, *J* = 5.0 Hz). ^13 ^C NMR (150 MHz, CDCl_3_): *δ* = 160.7, 155.2, 151,8, 147.6, 135.6, 134.6, 130.9, 130.8, 130.1, 124.5, 122.3, 109.6, 105.7, 103.5, 55.3, 49.9, 49.4, 47.1, 42.6; HRMS calcd for C_19_H_19_ClN_6_NaO_2_ [M + Na]^+^ 421.1156, found 421.1188.

##### 4.1.6.27. 1-(3-Chlorophenyl)-5-(4-(3,5-dimethoxyphenyl)piperazine-1-carbonyl)-1H-tetrazol (6–27)

Light yellow Solid; yield: 70%; Mp: 132.4–134.1 °C; ^1^H NMR (600 MHz, CDCl_3_): *δ* = 7.63 (1H, s), 7.55 (1H, m), 7.50 (2H, m), 6.08 (3H, s), 3.93 (2H, t, *J* = 5.2 Hz), 3.78 (8H, s), 3.26 (2H, t, *J* = 4.9 Hz), 3.20 (2H, t, *J* = 5.2 Hz). ^13 ^C NMR (150 MHz, CDCl_3_): *δ* = 160.5 (2 C), 154.1, 151.3, 146.6, 134.5, 133.6, 129.9, 129.7, 123.5, 121.3, 94.8 (2 C), 91.7, 54.3 (2 C), 48.9, 48.4, 45.9, 41.5; HRMS calcd for C_20_H_22_ClN_6_O_3_ [M + H]^+^ 429.1442, found 429.1449.

##### 4.1.6.28. 1-(2-Bromophenyl)-5-(4-(3,5-dimethoxyphenyl)piperazine-1-carbonyl)-1H-tetrazol (6–28)

Yellow Solid; yield: 80%; Mp: 71.1–72.4 °C; ^1^H NMR (600 MHz, CDCl_3_): *δ* = 7.74 (1H, dd, *J* = 7.9 Hz, *J* = 1.0 Hz), 7.56 (2H, m), 7.47 (1H, m), 6.09 (2H, s), 6.08 (1H, d, *J* = 2.0 Hz), 4.10 (2H, t, *J* = 5.0 Hz), 3.86 (2H, t, *J* = 5.4 Hz), 3.78 (6H, s), 3.29 (2H, t, *J* = 5.0 Hz), 3.24 (2H, t, *J* = 5.4 Hz). ^13 ^C NMR (150 MHz, CDCl_3_): *δ* = 160.5 (2 C), 153.5, 151.5, 147.9, 132.6, 132.5, 131.4, 127.8, 127.7, 118.7, 94.7 (2 C), 91.5, 54.3 (2 C), 48.8, 48.1, 46.1, 41.4; HRMS calcd for C_20_H_22_BrN_6_O_3_ [M + H]^+^ 473.0937, found 473.0954.

##### 4.1.6.29. 1-(3-Bromophenyl)-5-(4-(3,5-dimethoxyphenyl)piperazine-1-carbonyl)-1H-tetrazol (6–29)

White Solid; yield: 52%; Mp: 127.4–129.2 °C; ^1^H NMR (600 MHz, CDCl_3_): *δ* = 7.78 (1H, t, *J* = 1.8 Hz), 7.70 (1H, d, *J* = 8.3 Hz), 7.54 (1H, d, *J* = 7.9 Hz), 7.44 (1H, t, *J* = 8.3 Hz), 6.09 (3H, s), 3.95 (2H, s), 3.78 (8H, s), 3.27 (2H, t, *J* = 5.4 Hz), 3.21 (2H, t, *J* = 5.4 Hz). ^13 ^C NMR (150 MHz, CDCl_3_): *δ* = 161.6 (2 C), 155.1, 152.2, 147.6, 134.7, 133.8, 131.0, 129.7, 127.3, 122.8, 95.9 (2 C), 92.8, 55.3 (2 C), 50.0, 49.5, 47.0, 42.6; HRMS calcd for C_20_H_21_BrN_6_NaO_3_ [M + Na]^+^ 495.0756, found 495.0784.

##### 4.1.6.30. 1-(4-Bromophenyl)-5-(4-(3,5-dimethoxyphenyl)piperazine-1-carbonyl)-1H-tetrazol (6–30)

Brown Solid; yield: 72%; Mp: 122.4–123.2 °C; ^1^H NMR (600 MHz, CDCl_3_): *δ* = 7.62 (2H, d, *J* = 8.6 Hz), 7.40 (2H, d, *J* = 8.6 Hz), 6.01 (3H, s), 3.85 (2H, t, *J* = 5.7 Hz), 3.71 (8H, s), 3.19 (2H, t, *J* = 5.3 Hz), 3.14 (2H, t, *J* = 5.2 Hz). ^13 ^C NMR (150 MHz, CDCl_3_): *δ* = 160.5 (2 C), 154.6, 152.1, 146.5, 131.9 (2 C), 131.6 (2 C), 124.7, 116.0, 94.9 (2 C), 91.2, 54.3 (2 C), 48.9, 48.4, 46.0, 41.6; HRMS calcd for C_20_H_21_BrN_6_NaO_3_ [M + Na]^+^ 495.0756, found 495.0804.

##### 4.1.6.31. 1-(2-Methylphenyl)-5-(4-(3,5-dimethoxyphenyl)piperazine-1-carbonyl)-1H-tetrazol (6–31)

Yellow Solid; yield: 85%; Mp: 74.1–76.0 °C; ^1^H NMR (600 MHz, CDCl_3_): *δ* = 7.48 (1H, t, *J* = 7.6 Hz), 7.40 (1H, d, *J* = 7.8 Hz), 7.36 (1H, t, *J* = 7.4 Hz), 7.26 (1H, s), 6.08 (3H, s), 3.95 (2H, t, *J* = 5.7 Hz), 3.83 (2H, t, *J* = 5.0 Hz), 3.77 (6H, s), 3.23 (2H, t, *J* = 5.2 Hz), 3.20 (2H, t, *J* = 5.2 Hz), 2.11 (3H, s). ^13 ^C NMR (150 MHz, CDCl_3_): *δ* = 160.5 (2 C), 153.7, 151.4, 147.7, 133.8, 132.1, 130.4, 130.2, 125.9, 125.5, 94.8 (2 C), 91.6, 54.3 (2 C), 48.9, 48.3, 45.9, 41.4, 16.5; HRMS calcd for C_21_H_24_N_6_NaO_3_ [M + Na]^+^ 431.1808, found 431.1849.

##### 4.1.6.32. 1-(3-Methylphenyl)-5-(4-(3,5-dimethoxyphenyl)piperazine-1-carbonyl)-1H-tetrazol (6–32)

Brown Solid; yield: 69%; Mp: 76.6–78.3 °C; ^1^H NMR (600 MHz, CDCl_3_): *δ* = 7.43 (2H, m), 7.34 (2H, m), 6.09 (3H, s), 3.93 (2H, s), 3.78 (6H, s), 3.70 (2H, s), 3.24 (2H, t, *J* = 5.4 Hz), 3.15 (2H, s), 2.45 (3H, s). ^13 ^C NMR (150 MHz, CDCl_3_): *δ* = 160.6 (2 C), 154.7, 151.2, 146.6, 139.2, 132.6, 130.5, 128.5, 123.5, 119.9, 95.0 (2 C), 91.9, 54.3 (2 C), 49.0, 48.5, 45.7, 41.3, 20.4; HRMS calcd for C_21_H_24_N_6_NaO_3_ [M + Na]^+^ 431.1808, found 431.1854.

### 4.2. Biological evaluation

#### 4.2.1. Cell culture

The human gastric adenocarcinoma SGC-7901 cells, lung adenocarcinoma A549 cells and cervical carcinoma HeLa cells were cultured in RPMI-1640 medium containing 10% FBS, 100 U/mL streptomycin and 100 U/mL penicillin at 37 °C in a humidified atmosphere containing 5% CO_2_. All cell lines were purchased from the American Type Culture Collection (ATCC, Manassas, VA).

#### 4.2.2. In vitro *antiproliferative activity*

The antiproliferative activity assay *in vitro* was carried out referring to the previously reported method^22^.

#### 4.2.3. Effect on tubulin polymerisation

The tubulin polymerisation assay was performed by using the commercial tubulin polymerisation assay kit (Cytoskeleton-Cat.#BK011P) referred to the protocol of manufacturer^23^.

#### 4.2.4. Analysis of immunofluorescence staining

Immunofluorescence staining studies were investigated using the reported method^24^^,^^25^.

#### 4.2.5. Cell cycle analysis

Cell cycle analysis assay was followed the procedure of relevant report^26^.

### 4.3. Molecular modelling

Molecular modelling studies. Molecular docking was carried out by CDOCKER programme of Discovery Studio 3.0 software (PDB: 3HKC). The 3 D structure of 3HKC in docking study was downloaded from Protein Data Bank. The docking poses were selected according to previous studies^7^.
